# Topics of Public Interest

**Published:** 1914-08

**Authors:** 


### TOPICS OF PUBLIC INTEREST.

---

### Information Regarding Red Cross Organizations

Under orders from the War Department, the Red Cross may be called upon to furnish the following classes of individuals as needed.

1. Physicians and Surgeons.
2. Dentists.
3. Pharmacists.
4. Nurses.
5. Clerks.
6. Cooks and operating room attendants.
7. Litter bearers, drivers, teamsters, farriers and horse shoers.
8. Laborers.

Persons desiring to serve the Red Cross should indicate which of the following classes they desire to be considered under:

Class (a) Those willing to serve wherever needed.

Class (b) Those willing to serve in home country only.

Class (c) Those willing to serve at place of residence only.

The Red Cross does not desire the service of persons in the zone of operation under the age of twenty years nor over fifty years of age.

Under Class (a) the Red Cross is called upon to organize Field Columns, Hospital Columns, Supply Columns, and Information Bureaus.

The Field Columns require the following personnel:

1 Director. 4 Assistant Directors. 6 Section Chiefs. 16 Assistant Section Chiefs. 64 Men.

These organizations may be divided into four sections. Two of the above Section Chiefs are available for staff duty; that is, the keeping of records and the conduct of the supply department. All Directors and Assistant Directors must be qualified practitioners in good standing. The Red Cross prefers that such men should receive the endorsement of the "Committee on Red Cross Medical Work" of their local city, district or county medical society.

The Hospital Columns consist of:

1 Director, 3 Assistant Directors, 2 Section Chiefs, 6 Chief Nurses, 45 Nurses, cooks, ward orderlies, laborers, etc.

These organizations are divisible into three sections. The two section chiefs are required to keep the records and have charge of the supply service for the Column.

All Directors, Assistant Directors and Staff Physicians must be qualified physicians in good standing and should obtain the endorsement of the "Committee on Red Cross Medical Work" of their local city, district or county medical society, as indicated for the Field Columns.

Supply Columns usually consist of one Director and a number of pharmacists or drug clerks, teamsters, laborers and handlers, and will be attached to the supply depot of the army and navy departments as required.

The Red Cross personnel who have not had typhoid fever, and are under 45 years of age, will be required to receive antityphoid prophylaxis before proceeding to the front, and they must also submit evidence of successful vaccination.

The baggage of each individual when proceeding to the zone of operation should be reduced to a minimum, not to exceed 60 lbs., and should include a good mosquito net of fine mesh.

Red Cross organizations, when proceeding to the zone of operations and returning therefrom, will be transported and subsisted at the expense of the Government, and the Red Cross will pay adequate salaries for the various positions, which will be approximately as follows, including rations:

| | | |
|---|---|---|
| Directors | $250.00 | per month. |
| Assistant Directors and Staff Surgeons | 166.00 | " " |
| Section Chiefs | 60.00 | " " |
| Assistant Section Chiefs | 40.00 | " " |
| Other personnel | 18.00 | " " |

Pay of nurses, (female) will be the same as that of the regular Army Nurses Service.

The Uniform of the Red Cross will be a norfolk jacket and breeches of forest green; canvas leggings and campaign hat. The Red Cross brassard will be worn upon the left arm.

With the first month's salary to those below the grade of Staff Physician, the Red Cross will allow the sum of $17.00 to assist in defraying the cost of the uniform which thereafter must be obtained at the expense of the individual.

The Red Cross cannot do more at present than obtain lists of individuals who are willing to serve, pending a request by the War Department for actual assistance, and this information is intended to assist such individuals to prepare themselves for possible duty. The names and addresses of persons volunteering their services will be placed on file. All nurses, however, will be furnished by the Nurses Department of the Red Cross.

The information herein contained is general; further information can be obtained by application to this office.

ROBERT V. PATTERSON,
Major, Medical Corps, U. S. Army,
Chief, Bureau of Medical Service, A. R. C.

---

**Income Tax.** The total tax collected by the U. S. for the last ten months of 1913, was over 71 millions, of which N. Y., Pa., and Ill. paid nearly 38 million. N. Y. State 12½ million of slightly over 28 million personal tax and nearly 10 million of the total 43 million corporation tax. The Wall Street district paid nearly 8 million personal tax and nearly 6 million corporation tax. Buffalo paid nearly a million corporation and excise tax, over half a million personal tax.

---

**Physicians Compensation Under Workingman's Compensation Law.** Dr. T. H. McKee of the committee of the State Medical Society, advises physicians to make no contract until the committee arrives at an amicable adjustment of fees with the accident insurance companies of the state.

---

**Christian Science Verdict.** A lower court having found Willis Vernon Cole of N. Y., guilty of practicing medicine without a license, for having charged a fee for prayers for a patient, has been sustained by a higher (appellate?) court. Justice Laughlin held that the commercialized use of prayer was not the practice of the religious tenets of a church. Justice Cole concurred in the general verdict but held that a charge might properly have been made if the healer had practiced at the home of the patient or at a Christian Science Church. The case will probably be appealed.

---

**The Rollier Treatment,** including the sun bath, as practiced in Switzerland, will be carried out at the J. N. Adam Memorial Hospital of Perrysburg, in incipient tuberculous cases. Dr. John A. Ragone who has studied under Dr. Rollier and other European paediatrics, will have charge. Children may be referred to the Tuberculosis Dispensary 175 E. Swan Street, Buffalo, on Saturdays, between 10 and 12 and on Mondays between 3 and 5.

**New York City Cases Suspected of Malignancy** will be subjected to the diagnostic test by the N. Y. State Institute of Malignant Disease, situated in Buffalo.

---

**The Bingham State Hospital for the Insane** is being investigated regarding charges of tainted meat, etc.

---

**The Panama-Pacific Exposition,** to be held in San Francisco, next year, announces the following matters of interest to the medical profession:

Army wagons, tents and equipment used in the Civil War are expected to form a section of the exhibit being prepared by the War Department. Side by side with the modern paraphernalia of war, will be shown models, and in many cases originals, of the equipment of bygone days. A complete field hospital in operation throughout the period of the Exposition will be another feature of the exhibit of Uncle Sam's fighting forces.

The American National Red Cross will occupy a large space in the Palace of Liberal Arts, and the exhibit will include a panorama of Messina, Italy, showing in a vivid fashion the relief work of the Red Cross during the disaster which overtook the Sicilian city. The background will show mountains and the ruined city, and the foreground the American town which was built for the refugees out of the funds contributed to the Red Cross. The equipment for relief on battlefields will be shown in all of its details, and there will be various exhibits showing the work done during the Ohio floods of 1913, and numerous other object lessons in the splendid work of which the Red Cross is capable and is ready to undertake at short notice.

---

**Obesity Nostrums.** The U. S. Dept. of Agriculture has been investigating these frauds and has published a warning for the laity. In several instances these enterprises have been denied the use of the mails.

---

**The Wellcome Historical Medical Museum,** which was founded by Mr. Henry S. Wellcome in connection with the Seventeenth International Congress of Medicine, was reopened on May 28th, as a permanent institution in London. It is now known as the "Wellcome Historical Medical Museum" and is open daily from 10 a. m. to 6 p. m., closing at 1 p. m. on Saturday; entrance 54A, Wigmore Street, Cavendish Square, W. Since closing last October the collections in the Museum have been considerably augmented and entirely rearranged. Many objects of importance and interest have been added, which it is hoped will increase the usefulness of the Museum to those interested in the History of Medicine. Members of the medical and kindred professions are admitted on presenting their visiting cards. Tickets of admission may be obtained by others interested in the History of Medicine on application to the Curator, accompanied by an introduction from a registered medical practitioner. Ladies will be admitted, **only** if accompanied by a qualified medical man.

---

**Sex Education.** The National Educational Institution has declared against the teaching of this subject in the public schools and advises that it be left to parents.

---

**Expert on Sanitation.** The U. S. Civil Service Commission announce a competition open to males and females, to fill a vacancy in the Children's Bureau of the Dept. of Labor at Washington, at a salary of $2800. Applications must be filed complete, using form 304 and a special form for this examination, by Aug. 10. Blanks may be secured of the Commission, of the Custom House of N. Y. City and at various other places throughout the country.

---

**Cattaraugus Co. Tuberculosis Hospital.** An attempt to defeat the erection of such a hospital was voted down on July 10, by a vote of 29 to 3 of the supervisors.

---

**American Medicine Gold Medal.** The trustees announce that the medal for 1914 has been conferred upon Dr. George W. Crile of Cleveland, as the American physician who has performed the most conspicuous service in the domain of medicine during the past year. With this decision, the profession will unanimously concur.

---

**Full Time Medical Teaching.** The General Education Board, Whitehall, Bldg., N. Y. City, announces that it has transferred securities amounting to one and a half million dollars to the Medical School of Johns Hopkins University, to constitute the Wm. H. Welch endowment. Theodore C. Janeway of N. Y., formerly of Columbia, will be Professor of Medicine; Wm. S. Halsted of Baltimore will continue in the chair of surgery; John Howland, formerly of Washington University, St. Louis, will be Professor of Paediatrics, having already been in charge of the Harriet Lane Home for Invalid Children, under the auspices of Johns Hopkins, for a year.

While Johns Hopkins will be the first medical school to be placed on a full-time basis in all departments, Washington University of St. Louis and the Yale Medical School have received, respectively, gifts of 750 and 500 thousand dollars, upon the understanding that their departments be reorganized on this plan.

Full-time employment of medical teachers is a phase of medical education of the utmost importance, involving far-reaching tendencies, both favorable and unfavorable. As we pointed out several years ago, very disagreeable problems in ethics, threatening professional harmony, will be done away with, when the medical teacher becomes solely a teacher. In the beginning, the medical school was frankly and mainly unobjectionably, commercial. Students bought and certain physicians sold medical instruction. So long as the goods were sold with due regard to the fitness of the recipient to use them in the best interests of the community and the sellers gave honest value in goods which legally and morally belonged to them to sell, no ethical problem presented itself. But, as matters developed, very serious ethical problems arose. In the first place, goods of adequate quality simply could not be sold at a price which the student could pay and which had become pretty firmly fixed by competition. In the second place, the publicity connected with the sale of the goods became a more important element than the direct financial compensation. Hostility inevitably developed between the medical teacher and the profession and all sorts of secondary evils arose. These evils have, fortunately been corrected to a large degree in advance of the establishment of medical teaching as a phase of state or municipal government or of private philanthropy on a large scale. Yet the establishment of medical teaching on the same ethical and economic basis as other teaching, remains a matter of the utmost importance.

On the other hand, certain draw-backs exist to the general establishment of full-time medical teaching. With three important schools thus established, there is bound to be a demand by several others for donations from a public already taxed to the utmost for the ordinary purposes of government and for a multitude of philanthropy. An adequate response can come only from sources that have been called tainted and which are, at least, somewhat liable to impose conditions or to imply obligations that may not be altogether desirable. With a considerable minority of medical schools endowed so as to be entirely relieved of the economic responsibilities of obtaining funds and acting as donors to medical students of values far in excess of their fees, there will enter the factor of giving something for nothing, which is always detrimental. Competition between philanthropy and business is always a menace to the latter. In this case, a large number of worthy institutions, ambitious to fulfill their responsibilities in the widest sense, will be placed in the equivocal position of requiring money, in large amount, to fulfill either the moral or, ultimately, the legal demands made upon them. We view with considerable apprehension, the placing of an individual or an institution in an unfavorable moral and legal light, simply for want of funds and especially for want of such an amount as he or it can reasonably expect to earn by means established by precedent as legitimate. Demands far less radical than will be made under the accepted standard of full-time teaching in all departments, have already killed a good many colleges. One or two have become institutions for the profession itself and thus will continue to fulfill a useful purpose; many that have been killed deserved to die; but there remain many whose demise, past or future, is to be regretted. A peasant expostulating against a high tax, exclaimed, "I must live!" "Really," replied the official responsible, "I see no necessity for it." It would be a sorry thing for a democratic profession in a democratic country to encounter this idea as a practical danger to the existence of any of its institutions.

A further draw-back to the general adoption of the plan of making medical teachers a class by themselves, is that the ultimate object of medical education is to train physicians. We make this claim advisedly and in spite of the somewhat supercilious attitude which certain censors of medical education have assumed with regard to the mere practice of medicine. Having been offered a career in scientific medicine not involving private practice, we feel that we can regard this matter impartially without going either to the extreme of "Old Doc." in his ignorant contempt for medical science, or to the opposite extreme of science and scholasticism. The medical student needs guidance from his teachers, not merely with regard to scientific and empiric facts in medicine but with regard to the economic and ethical problems which he is to encounter as a practitioner. Such guidance he can obtain only from men who have been through what he is about to enter. For the first few years of the full-time method of education, this lack will not be conspicuous. In another generation, it will have become a serious problem. In our observation, private practice is necessary not only to fit the teacher to be an advisor of prospective practitioners in economic and ethical matters but even to insure sound practical judgment in medical matters. We venture to prophecy that, if full-time medical instruction becomes a general custom, the profession will have to return to the old time system of preceptorship not merely in the nominal sense in which middle aged men remember it, but in the literal and practical sense of a couple of generations ago.

---

**The School of Commerce, Accounts and Finance of New York University** has issued a bulletin of its Division of Advertising. Day and evening sessions will be held. We note the absence of special instruction in medical advertising—a most important factor not only ethically as it appeals to the medical profession but considering the magnitude of the industries involved.

---

**Sterilization Laws.** Two U. S. District Judges have concurred in the decision of Judge Smith MacPherson that the Ohio sterilization law is unconstitutional, not only in involving a cruel and unusual punishment—formerly in vogue and discarded by practically all civilized governments—but in failing to provide for due process of law in the defense.

---

**A Site for a County Tuberculosis Hospital** has been selected three miles from Olean, on the line of the W. N. Y. & P Traction Co. The site contains ample springs and a natural gas well.

---

**The Hospital Ship Maine,** presented to the British Government by American women, at the time of the Boer War, was wrecked in a fog in the Firth of Lorne, June 17. The patients were transferred in small boats without loss.

---

**Largest Bequest to Medical Philanthropy.** James Campbell of St. Louis has made St. Louis University residuary legatee of his estate of 25 millions, for the establishment of a hospital and for the advancement of medical sciences.

---

**Quintuplets** were born to Rosa Corrada of Palermo, Italy, this spring, all living and well.

---

**Joined Twins Separated.** Drs. Mignot and Du Boucset of Paris, on July 16, separated twins born May 22 and joined in the lumbar region. The operation included a separation of the intestine for a length of 3 c.m.

---

**Sixth Plague Case.** The sixth case of bubonic plague in New Orleans was found July 18. The illness of Helen Soell, aged ten years, was diagnosed as plague and the child was removed to the isolation hospital.

